# Supplementary material for: Comparison of the performance of Aptima HIV-1 Quant Dx Assay with Abbott RealTime HIV Assay for viral load monitoring using plasma and Dried Blood Spots collected in Kenya
Source: PLoS One. 2022 Aug 22;17(8):e0269838. doi: 10.1371/journal.pone.0269838 (PMC9394820; doi:10.1371/journal.pone.0269838)
Supplement: Attachment — Submitted filename: Rebuttal notes Aptima Adult Paper May 6 2022.docx [file pone.0269838.s002.docx]

**Rebuttal notes**

Line 30: remove the word consenting—this is not needed in the abstract.

**The word “consenting” has been removed from line 30.**

I agree that line 33 is confusing—it could imply that DBS were made from the venous blood of some of the patients and from fingerstick from others, or it could imply that DBS were not made from the blood of all patients included, but the earlier sentence says that DBS were collected from all 1312.

**We have rewritten the methods section (lines 27-34) to read as follows:**

**This was a cross-sectional study of 2227 HIV infected adults visiting health facilities in Nairobi and Busia, Kenya. Each provided a venous blood sample; plasma was prepared from 1312 samples while paired DBS samples and plasma were prepared from the remaining 915 samples. The agreement between the Aptima assay and the Abbott RealTime HIV-1 Assay (Abbott RT) was analysed by comparing the HIV-1 viral load in both assays at the medical decision point of 1000 copies/mL. To assess stability of HIV-1 RNA in DBS, viral load in DBS spotted on day 0 were compared with that from the same DBS card after 21 days of storage at room temperature.**

Suggested word changes starting at line 33: “…these patients. Agreement between the Aptima…”. I would suggest dropping the word total throughout because it does not add further understanding of what was analyzed. However, it was used in the previous publication, so keeping it would be consistent.

**We have deleted several “total” words throughout the manuscript. We have left a few of the words where the sense would be lost if we deleted them.**

The authors should mention their previous study in the introduction (not just as a citation), that the current study is an extension of that one with more data, and especially to note that fingerstick and venous DBS were compared in the previous study so they didn’t need to do that here. In the discussion (lines 326-327), they should acknowledge their previous study is their own and that the current study extends from the previous one.

**We have rewritten the paragraph that starts from line 85 to capture these details as follows:**

**In a previous study, we investigated the performance of the Aptima HIV Quant Dx assay using fingerstick and venous dried blood spots prepared under field conditions, although we did not compare HIV-1 VL in Aptima with those in other assays using DBS. In fact, whereas multiple studies compared viral load results of paired DBS and plasma samples at the medical decision point of 1000 copies/ mL by testing both sample types in Aptima [21-23, 31], there is no published information comparing the performance of DBS specimens tested in Aptima and other assays at the medical decision point (MDP) of 1000 copies/mL.**

The authors need to add the specific number of samples used in this study in the methods section. This includes the number people with both plasma and DBS, the number with only plasma, and the number with only DBS.

**We have rewritten the methods section to make sure this is very clear, as follows:**

**Whole blood was collected from 2227 participants by phlebotomy, shipped to KEMRI HIV laboratories within 6 hours of collection, and processed within 12 hours of receipt. A total of 1312 samples provided only plasma. Paired DBS and plasma samples were prepared from a further 915 venous samples. To prepare plasma, the whole blood samples were centrifuged at 1,100g for 10 minutes within 24 hours of collection, and the plasma was stored at -80^0^C. To prepare DBS, 70µl of venous blood was spotted in each of ten spots (2 DBS cards) per patient. The DBS samples were allowed to dry overnight. The first DBS card for each patient was used for day 0 testing, while the DBS cards for the 21-day time point were packaged with desiccants and stored at room temperature. All samples were de-identified prior to Aptima testing.**

Line 283: change wording to “HIV viral load >1000 copies/mL in Aptima but <1000 copies/mL in Abbott RT”

**The word “but” has been added, apologies for the oversight in this sentence.**

Lines 305-324: This paragraph should mostly move to the results section because it describes a new comparison of the data. In the Discussion, there should be a new paragraph about this analysis to put the results into context.

**The results had already been presented in the results section, and for this reason we have carefully revised the paragraph to lighten it while still retaining its meaning, from lines 317-329**

Reviewer #1: Review of responses to reviewers’ comments

The major issues to address are the inconsistent numbers throughout, the lack of replicate testing, and the higher rate of quantitative values from DBS than from plasma. These and other issues are detailed below:

a. Corrected the inconsistent in N from 439 to 436 for quantified plasma samples throughout the document:

**n is now corrected to 436 where appropriate**

Figure 1 legend still states 439 samples.

**This has been corrected. Line 234 now reads: Figure 1a: HIV viral load comparison for 436 plasma samples tested in Aptima and Abbott RT assays**

c. The number of quantifiable results in plasma and DBS sample sets are different because not all plasma and DBS were prepared from the same patients. Blood samples from only a subset of patients were used to prepare plasma and DBS for testing in the two assays. The study also included some DBS samples that did not have corresponding plasma samples and vice versa.

**In the methods section, we have clarified the exact number of samples of each type used in the study.**

**Please see lines 127-136**

Line 32 of the revised manuscripts states that “Both plasma and DBS samples were collected from each of 1312 adults who participated.” but line 35 DBS was prepared using venous blood from a subset of these patient.” which is conflicting.

**This is our error. We have rewritten lines 27 to 31 to read as follows:**

**This was a cross-sectional study of 2227 HIV infected adults visiting health facilities in Nairobi and Busia, Kenya. Each provided a venous blood sample; plasma was prepared from 1312 samples. Paired DBS and plasma samples were prepared from the remaining 915 samples.**

Make sure everything is the same between the text and the abstract and the figures (436 vs 439; r = 0.96
vs R = 0.92—need to check both the number and the capitalization for that one; r = 0.87 vs R = 0.8692
need to check number of significant digits and capitalization; mean bias for DBS storage = -0.154 vs -
0.15). Be consistent with your significant digits and with small case r throughout.
Changed correlation coefficient from “R” to “r” in the document

**This has now been done throughout the document.**

Still capitalized in line 231 of the revised manuscript, R of 0.92 is different to the R= 0.9589 on the figure.

**We have corrected this error in the manuscript, and resolved capitalisation around *r*.**

Line 184: discordance rates not shown in Table 1 but can be obtained with the positive agreement values in Table 1, so re-write this statement. This paragraph (lines 184-191) is difficult to understand in terms of useful results. It is useful to the reader to know how many of the discordant results were due to higher detection in one assay vs the other, but the value of the rest of the “results” is less clear. What is the goal of reporting that 17 of the discordant samples (Abbott higher than Aptima in DBS) had corresponding plasma VL that were higher than 3631cp/mL? Is that VL cutoff a magic number for these assays? There is no mention of these “results” in the discussion, providing no context for why this “result” should be noted.

**We have removed the reference to Table 1 from the statement of discordant rates.
The purpose of pointing out that 31 samples with discordant DBS results at MDP of 1000 c/mL had plasma results <3631 c/mL is to demonstrate that these samples may flip above and below a 1000 c/mL in different replicates because the HIV concentration is close to a 1000 c/mL. We have also added a couple of sentences under the discussion to explain the DBS discordants to link with the results in this section.**

There is still no indication of why 3631 cp/mL (3,56 log10) specifically was chosen as a cut –off. Using a range of 2,5 – 3,5 log10 incorporating the 0,5 log10 accepted variation in VL assays, would be relevant to justify the flip argument suggested.

**Your point is well noted. We agree that there is nothing special about the 3631cp/ml. We have rewritten the sentence (lines 326-329) as follows:**

**The remaining 14 of the 31 patients with Aptima DBS VL <3 log c/mL and Abbott DBS VL>3 log c/mL had plasma VL >3.00 log copies/mL in both Aptima plasma and Abbott plasma suggesting that these were upward misclassifications by Abbott DBS.**

Major points

1. It is not clear at abstract’s reading that the authors were working on DBS prepared from venous blood and not from finger-stick. The number of quantifiable samples is different in abstract 436 and results 439.

**We have added a statement in the methods that venous DBS was used for this study. Please see line 23.**

**We have corrected the number of quantified samples to 436, and indicted that venous DBS was used, throughout the document.**

Figure 1 still states 439 samples.

**We have corrected this to 436 samples in Fig. 1.**

6. 439 quantifiable plasma VL but 636 quantifiable fresh VB DBS: this discrepancy is not discussed.

**The 436 quantifiable plasma samples were prepared from venous blood taken from 1312 patients. The 636 quantifiable DBS samples were prepared from a further group of (915) participants, who also provided plasma.**

**For that reason the yields are not comparable.**

Does this explanation provided by the authors indicate that the discordant DBS samples were only found in the subgroup that had both plasma and DBS samples types?

**As clarified, there were 915 paired DBS and plasma samples. The 49 discordant DBS samples are a subset of the 915 samples.**

There are 197 more quantifiable DBS results than plasma which is unusual in a comparison of these sample types particularly since the DBS samples were made from the 1312 enrolled patients and has a higher limit of quantification than plasma. Is this related to the run failure? It is important to clarify the reason for the difference.

**These results arise from different sets of participants. So that comparison does not arise. Please see lines 127-136.**

**Whole blood was collected from 2227 participants by phlebotomy, shipped to KEMRI HIV laboratories within 6 hours of collection, and processed within 12 hours of receipt. A total of 1312 samples provided only plasma. Paired DBS and plasma samples were prepared from a further 915 venous samples.**

**The 49 discordant DBS samples were part of the 915 paired DBS and plasma samples tested.**

Minor points

3. In the methods, a flow chat of tested samples is lacking.

**A total of 1312 samples provided only plasma. Paired DBS and plasma samples were prepared from a further 915 venous samples. Since there was no further dichotomy, a flow chart was not necessary.**

It would still have been easier to understand the sample numbers of the enrolled samples had been described more clearly in the text or in the flow chart suggested above; according the total number of samples enrolled, number of plasma only, both plasma and DBS, and DBS only. The sample numbers are raised several times in the review because it not defined clearly enough in the methods and results.

**We have extensively rewritten this section to make it as clear as possible. Please see lines 179-185.**

Second review

Line 24 What criteria were used to define high throughput?

**There are no universally agreed criteria for defining high throughput. In our setting, we consider low throughput to mean <96 tests in 24 hours, medium throughput to mean 97-480 tests in 24 hours and high throughput to be >481 tests in 24 hours.**

Line 112 Stating that some DBS samples did not have paired plasma samples under the study design heading suggests that was intentional. Is this correct?

**This was our mistake. (In actual fact, this error occurred out of confusion from: 258 of our DBS samples had accompanying fingerstick samples while the rest did not, but that data is reported elsewhere already).**

**As stated in the manuscript, a total of 1312 samples provided only plasma. Both plasma and paired DBS samples were prepared from a further 915 venous samples.**

Lines 223 – 225 What happened to the other 10 upward misclassified DBS samples?

**We have revised this section so that the discussion does not mislead, from lines 335-346.**

Line 321 – Does this include the Aptima plasma? it is not clear.

**The upward misclassification was seen only in Aptima DBS, not Aptima or Abbott plasma or Abbott DBS.**

Line 284 – No discussion about possible under quantification by the Realtime assay.

**The discussion around this finding has now been provided, lines 331-342**

A general review of the grammar and formatting is required including spacing, punctuation and duplication of a few words.

**We have used the grammar tool to resolve spacing challenges and correct grammatical errors.**
